# Label-Free Comparative Proteomics Analysis Revealed Heat Stress Responsive Mechanism in *Hypsizygus marmoreus*

**DOI:** 10.3389/fmicb.2020.541967

**Published:** 2021-01-05

**Authors:** Lili Xu, Lizhong Guo, Hao Yu

**Affiliations:** Shandong Provincial Key Laboratory of Applied Mycology, College of Life Sciences, Qingdao Agricultural University, Qingdao, China

**Keywords:** *Hypsizygus marmoreus*, proteomics, label free quantification, heat stress response, heat stress proteins

## Abstract

Heat stress is an important adverse environmental stress that influences the growth and development of *Hypsizygus marmoreus* (white var.). However, the molecular basis of heat stress response in *H. marmoreus* remains poorly understood. In this study, label-free comparative proteomic technique was applied to investigate global protein expression profile of *H. marmoreus* mycelia under heat stress. Confocal laser scanning microscope observation revealed that mycelia underwent autolysis and apoptosis under heat stress. Autolysis was mediated by upregulating the expression of cell wall degradation enzymes and inhibiting cell wall synthesis enzymes, and apoptosis might be induced by ROS and activation of caspases. TBARS analysis indicated that ROS was accumulated in *H. marmoreus* mycelia under heat stress. *H. marmoreus* induced antioxidant defense system by upregulating the expression of catalases, superoxide dismutases and peroxidases to prevent oxidative damage. MAPK cascade was found to be involved in heat stress signal transduction. The stress signal induced a ubiquitous defense response: inducible expression of different kinds of heat shock proteins. Trehalose synthesis enzymes were also upregulated, suggesting the accumulation of stress protector trehalose under heat stress. Besides, upregulated proteasome was identified, which could prevented the accumulation of non-functional misfolding proteins. To satisfy ATP depletion in heat response cellular processes, such as ROS scavenging, and protein folding and synthesis, enzymes involved in energy production (carbon metabolism and ATP synthesis) system were upregulated under heat stress. Taken together, these findings improve our understanding of the molecular mechanisms underlying the response of heat stress in *H. marmoreus*.

## Introduction

*Hypsizygus marmoreus* is one of the most popular industrialized cultivation mushrooms in East Asia, including China, Korea and Japan (Qiu et al., 2013; Chauhan et al., 2017; He et al., 2019; Wu et al., 2019). It draws our attention due to its desirable, mildly sweet nutty flavor, unique texture and medicinal properties (Chen et al., 2018; Liu et al., 2018a; Shah et al., 2018). As a result, the number of *H. marmoreus* cultivation factories increased gradually in different regions of the world, especially in China. *H. marmoreus* is the third wildly cultivated industrialized cultivated mushroom in China now behind only *Flammulina velutipes* and *Pleurotus eryngii* (Li et al., 2019). *H. marmoreus* is a low-temperature fruiting mushroom. The mycelia grow vegetatively at 23°C to 25°C and transform into fruiting process at 13°C to 17°C. The fruiting process of *H. marmoreus* is sensitive to temperature, and high temperature can lead to the death of mycelia and malformation of the fruiting body (Zhang et al., 2019; Xu et al., 2020). Therefore, thermotolerance is important for *H. marmoreus* during both vegetative growth and fruiting-body formation. Previous studies on *H. marmoreus* have mainly focused on cultivation, nutrition and bioactive compounds extracted (Mleczek et al., 2018; Saito et al., 2018; Oliveira et al., 2019; Tsai et al., 2019), but little is known about thermotolerance of this mushroom.

Heat stress is an important adverse environmental stress that influences all levels of biological organization including the growth and development of mushrooms. Heat stress can inhibit the mycelia growth and induce the autolysis and apoptosis of mushroom mycelia. Proteins which promote the natural degradation of cell wall and cytoplasm were upregulated under heat stress (Huang et al., 2011), and nuclear condensation and DNA fragmentation could be observed (Song et al., 2014). The cytosolic reactive oxygen species (ROS) concentration of mushroom mycelia significantly increased under heat stress leading to cell membrane peroxidation, protein oxidation and DNA damage (Kong et al., 2012a; Ghani et al., 2017; Lei et al., 2019). Heat treatment could also affect the intracellular and extracellular metabolites of mushroom mycelia, such as ganoderic acid and volatile components (Tereshina et al., 2011; Liu et al., 2018c; Zhang et al., 2019). To counteract the detrimental effects of heat, mushroom cells evolved multiple adaption systems by changing proteins expression including (1) heat stress signal responding transduction system (Guo et al., 2016; Loshchinina and Nikitina, 2016; Zhang et al., 2016; Liu et al., 2018b); (2) the induction of heat stress factors and heat shock proteins (HSPs) (Lee et al., 2006; Kurahashi et al., 2014; Zhang et al., 2016; Chen et al., 2017; Wang et al., 2018a); (3) ROS burst and significant antioxidant accumulations (Chen et al., 2017; Wang et al., 2017; Liu et al., 2018c); (4) anabolism inhibition and catabolism activation, such as trehalose accumulation (Kong et al., 2012b; Liu et al., 2016, 2019; Chen et al., 2017). Recently, the protection effect of antioxidant kojic acid in *H. marmoreus* mycelia damage was studied (Zhang et al., 2017). Kojic acid could promote mycelial regeneration by activating a stress signal and enhancing the activity of enzymes involved in ROS scavenging. Zhang et al. (2019) reported that heating could significantly enhance the color difference, and reduce all texture parameters as well as polyphenol oxidase and peroxidase activities in *H. marmoreus*. However, in general, information about heat stress response mechanism in *H. marmoreus* is quite limited. To understand the modulation mechanism of heat stress response in *H. marmoreus*, a detailed study at the proteomics level is necessary.

Recent developments in sequencing and proteomics technology and the increasing availability of genomic information have allowed for the successful use of omics study approaches to examine global mRNA/protein expression pattern and metabolites investigating mechanisms of organisms response to abiotic stresses (Bai et al., 2014; Zhang et al., 2015, 2017; Fu et al., 2016; Ma et al., 2018). Based on data gathered from proteomics, transcriptomics or metabonomics analysis, a number of physiological processes such as induction of ROS response and scavenging systems and HSPs have been proposed to be involved in mediating heat stress response (Huang et al., 2011; Chen et al., 2017; Qiu et al., 2018; Wang et al., 2018b,c; Zou et al., 2018). Recently, a RNA-sequencing study of *Pleurotus ostreatus* under high temperature conditions were reported (Wang et al., 2018c; Zou et al., 2018). A total of 20 *MYB* transcription factors were identified across the genome of *P. ostreatus*, and the expression pattern of *MYB* genes under heat stress were studied. *MYB* genes were up-regulated under heat stress, indicating they play important roles in heat stress response in *P. ostreatus* (Wang et al., 2018c). In another study, an iTRAQ-based quantitative proteomic analysis identified 204 differentially expressed proteins during heat stress and/or the recovery phase in *P. ostreatus*. This study showed the essential role of mitogen-activated protein kinase (MAPK)-pathway, antioxidant enzymes, HSPs, and glycolysis in heat stress adaptation and recovery (Zou et al., 2018). Qiu et al. analyzed the change of extracellular metabolites of *P. ostreatus* under heat stress. A total of 144 differential metabolites were identified, including 84.4% up-regulated. The randomly selected upregulated metabolites could promote the mycelial growth and conidial germination of mushroom pathogenic microorganism *Trichoderma asperelum*, which provided a first view that high temperature affected the resistance to biotic disease (Qiu et al., 2018). Wang et al. analyzed the mRNA and protein expression pattern of a heat-tolerant and heat-sensitive strains of *Lentinula edodes* under heat stress, revealing that HSPs and tryptophan as well as IAA metabolism pathway played important roles in thermotolerance (Wang et al., 2018b). *Ganoderma oregonense* transcriptional profiling in response to heat have also been monitored (Chen et al., 2017). This study provides the mechanism of nitric oxide, which could upregulate the expression of HSPs and ROS detoxification enzymes, in thermotolerance of *G. oregonense* at the gene expression level. In contrast to those mushrooms explored in abundant global analysis of heat stress response, *H. marmoreus* has not been subjected to any omics surveys for elucidating the mechanism of thermotolerance.

To elucidate temperature acclimatory responses in *H. marmoreus* mycelia, in the present study, the expression pattern changes in heat stress response of the industrialized cultivated *H. marmoreus* strain was examined. A label-free quantitative liquid chromatography-tandem mass spectrometry (LC-MS)/MS technique was used to assess proteome profile of *H. marmoreus* strains at normal temperature, elevated temperature and recovery stage after heat stress to clarify the adaptive mechanism. The analysis of heat-stress-related proteins will allow the determination of critical process-related proteins of this industrialized cultivated mushroom. The results not only facilitate a better understanding of molecular mechanisms at different subcellular levels of *H. marmoreus* mycelia response to heat stress but will also provide useful information for the rational design to improve thermotolerance of this important industrialized cultivated mushroom.

## Materials and Methods

### Strains, Cultivation Conditions and Heat Stress Treatment

*H. marmoreus* (white var.) strain G12 used in this study was provided by Shandong Provincial Key Laboratory of Applied Mycology. *H. marmoreus* mycelia were cultivated in potato dextrose agar (PDA: extract from 200 g potato, 20 g dextrose, 20 g agar) plate at 25°C in darkness as previously described (Wang et al., 2019; Xu et al., 2020). A total of 60 plates were divided randomly into three groups: Con group, HS group, and RHS group. Mycelia in Con group were cultured at 25°C for 16 days, and the mycelia on cellophane were collected as Con samples. Mycelia in HS group were cultured at 25°C for 15 days and then the plates were transferred to 37°C for 24 h. Mycelia for HS_8_ group were cultured at 25°C for 15 days and then the plates were transferred to 37°C for 8 h. Mycelia in RHS group were cultured at 25°C for 11 days, and then the plates were transferred to 37°C for 24 h. After heat treatment, mycelia in RHS group were transferred back to 25°C and cultured for 7 days. The mycelia sample of HS group and RHS group were collected as those of Con group. For each sample, mycelia from three plates (∼0.2 g) were collected and stored in one 2 mL centrifuge tube. All samples were immediately frozen in liquid nitrogen and stored at −80°C for further analysis.

### Microscopic Observation of *H. marmoreus* Mycelia

Nuclear and cell wall of the fungal cells were stained by carboxyfluorescein succinimidyl ester (CFSE) and Congo red simultaneously. The mycelia were stained with 1.0% (w/v) Congo red (dissolved in ddH_2_O) and 0.1 μg/mL of CFSE (dissolved in dimethyl sulfoxide) simultaneously for 10 min in darkness. Stained mycelia were washed with PBS for 5 min and observed with confocal laser scanning microscope (CLSM) (Leica TCS SP5 II, Bensheim, Germany).

### Thiobarbituric Acid Reactive Substances (TBARS) Measurement

TBARS was measured according to the previous studies described by Ghani et al. (2017) and Kong et al. (2012a). Briefly, 1 g mycelia were scraped off the cellophane, grounded into powder with liquid nitrogen and transferred into a 1.5 mL centrifuge tube. Then, 0.5 mL 5% (w/v) trichloroacetic acid was added and the mixture was incubated for 10 min in ice water bath. After centrifugation at 10,000 *g* for 10 min, the supernatant was mixed with 2 mL 0.67% (w/v) thiobarbituric acid. The mixture was incubated at 95°C for 10 min and centrifuged at 10,000 *g* for 10 min. The absorbance of the supernatant at 532 and 600 nm were measured using Cary 60 spectrophotometer (Agilent, CA, United States). All the tests were performed at least three repeats.

### Protein Extraction and Peptide Digestion

For protein extraction, 1 mL UA buffer (8 M urea, 0.1 M Tris-HCl pH 8.5) containing 1 mM phenylmethylsulfonyl fluoride and HaltTM Protease inhibitor Cocktail (Thermo Fisher, Shanghai, China) were added into the centrifuge tube. Mycelia was broken using TissueLyser II (QIAGEN, Hilden, Germany) at 150 Hz for 60 s. The cell extract was treated by ultrasonication for 24 s (on for 6 s, off for 15 s). Cell debris was removed by centrifugation at 12,000 × *g* for 10 min at 4°C, and the supernatant was transferred to a new tube. The protein concentration was measured with Micro BCA Protein Assay Kit (Thermo Fisher, Shanghai, China). Then 15 mg DTT was added into each sample, and the samples were incubated at 37°C for 1 h.

Enzymolysis was performed with FASP method according to the method described by Wiśniewski et al. (2009). 100 μg proteins for each samples were dissolved in 300 μL UA buffer and added into Pierce Protein Concentrators PES (10K MWCO, 0.5 mL) (Thermo Fisher, Shanghai, China). Ultrafiltration tube was centrifuged at 10,000 *g* for 30 min to remove the low molecular impurities. 100 μL UA buffer containing 50 mM iodoacetamide was added into the ultrafiltration tube and the tube was incubated at room temperature for 30 min. The buffer was removed by centrifugation and washed with 200 μL UA buffer for three times and 300 μL of 50 mM NH_4_HCO_3_ for two times. 2 μg Modified trypsin (Promega, Madison, WI, United States) in 100 μL of 50 mM NH_4_HCO_3_ was added into the ultrafiltration tube in mass proportion of 1:50 (enzyme/protein). Enzymolysis was performed with gentle shaking at 37°C for 12 h. Peptides were collected by centrifugation at 10,000 *g* for 15 min, and the residue peptide in the ultrafiltration tube was washed with 50 μL 50 mM NH_4_HCO_3_ for two times. The elutes were pooled and the salt was removed using Merck Millipore ZipTip C18 resin (Darmstadt, Germany). Peptide concentration was measured using Pierce Quantitative Colorimetric Peptide Assay (Thermo Fisher, Shanghai, China). Peptide samples were evaporated on a RVC 2-25 CD plus vacuum concentrator (Christ, Osterode am Harz, Germany), and stored at −80°C for further analysis.

### LC-MS/MS Analysis

Desalted peptides were reconstituted in 10 μL 0.1% formic acid. Label-free analysis was performed using an Nano-LC system coupled with Orbitrap Fusion^TM^ Tribrid^TM^ (Thermo Fisher, CA, United States). The MS instrument was operated in date-dependent acquisition mode, with full MS scans over a mass range of m/z 350–1500 with detection in the Orbitrap (120 K resolution) and with auto gain control set to 100,000. Different chromatographic gradient lengths from 60 to 240 min were tested for peptide separation. All gradient started at 5% (v/v) ACN (0.1% formic acid) and went up to 32% (v/v) ACN (0.1% formic acid). Two biological replicates for each group were prepared, and two technique replicates were performed for each sample.

### Peptides and Proteins Identification

The raw files were analyzed using Proteome Discoverer software suite version 2.0 (Thermo Fisher, CA, United States) against the *H. marmoreus* proteome database (proteome ID UP000076154) from Uniprot. Protein identification was supported by at least 2 unique peptides, and the false discovery rate was lower than 0.05.

### Bioinformatics Analysis

Protein with three missing value of one group was discarded. The *K*-nearest neighbors technique was used to estimate one or two missing values in one group. Data was normalized using median methods. Two sided *t* test was performed to detect fold change between each protein, the *p*-value from *t* test was adjusted by Benjamini-Hochberg (BH) adjusting methods. The corrected *p*-value BH < 0.01 was considered significant. The proteins were analyzed by gene ontology (GO) annotation derived from the gene ontology database^1^. Protein pathway were annotated by Kyoto Encyclopedia of Genes and Genomes (KEGG) database (Kanehisa et al., 2016). Protein-protein interaction network was analyzed using Serach Tool for the Retrieval of Interacting Genes/Proteins (STRING) platform and characterized using Cytoscape software (Shannon et al., 2003; Szklarczyk et al., 2015).

### RNA Extraction and Quantitative Real-Time PCR (qRT-PCR) Analysis

Twenty genes were selected to measure their mRNA expression. The cDNA sequences of these genes were retrieved from the whole genome sequence of *H. marmoreus* (white var.) strain 51987-8. Primers used in this study were listed in Supplementary Table 3. Considering that protein expression is lagging behind mRNA expression. Mycelia cultivated at 25°C for 15 days and treated at 37°C for 8 h (HS_8_) were also analyzed in qRT-PCR verification experiments. Total RNA of three samples, Con, HS_8_ and HS, was extracted from mycelia with RNAiso Plus Trizon reagent (Takara, Dalian, China) according to the manufacture’s instruction. RNA concentration was measured using NanoDrop One microvolume UV spectrophotometer (Thermo Fisher, CA, United States). cDNA library was prepared using RNA PCR Kit (AMV) Ver.3.0 (Takara, Dalian, China) according to the manufacture’s instruction. The reaction system was prepared using ChamQ Universal SYBR qPCR Master Mix (Vazyme, Nanjing, China) according to the manufacture’s instruction in a total volume of 15 μL for each tube. qPCR reactions were performed using QuantStudio 5 Real-Time PCR System (Thermo Fisher, CA, United States). Genes encoding 40S ribosomal protein (A0A369JF09) was used as internal control genes. Relative expression levels were calculated using the relative 2^–(ΔΔCt)^ method, and the expression level was normalized to the expression level of the control genes.

## Results

### Morphological Changes and Oxidative Damage in *H. marmoreus* Mycelia in Response to Heat Stress

**FIGURE 1:**
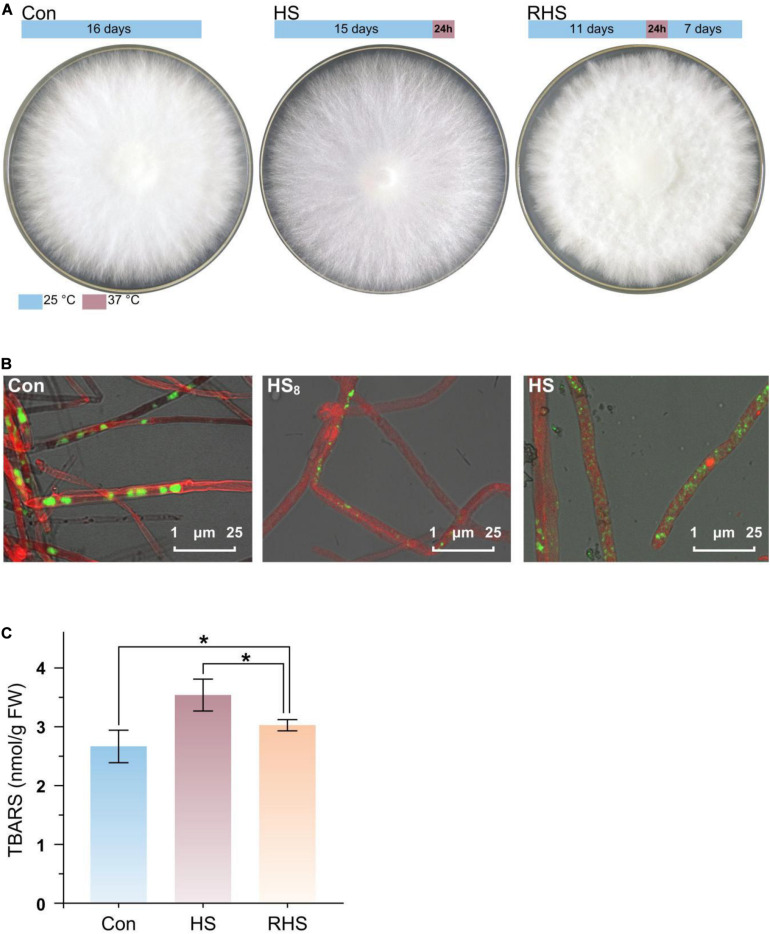
Mycelial morphology and TBARS concentration of *H. marmoreus* in response to heat stress. **(A)** Colony shape of three groups. The growth and heat treatment time and temperature were indicated with bar above the colony shape of each group. **(B)** Laser scanning confocal microscope photomicrographs of mycelia strained with CFSE and Congo red. Green, strained DNA; Red, strained cell wall. **(C)** TBARS concentration in mycelia from three groups. Each value is the mean from three parallel replicates ± SD. One-tailed *t* test, performed by SPSS 18.0 (Chicago, IL, United States), was used to calculate statistical significance. Symbols: *p < 0.05.

*H. marmoreus* mycelia were divided into three groups, mycelia grew at 25°C for 16 days (Con group), mycelia grew at 25°C for 15 days following a heat treatment at 37°C for 24 h (HS group), and mycelia grew at 25°C for 11 days following a 24 h heat treatment (at 37°C) and then 7 days recovery growth (at 25°C) (RHS group) (Figure 1A). Colonies from three groups just covered the entire plate after cultivation and exhibited different morphologies. Colonies from Con group produced vigorous aerial hyphae and the colonies were white. After heat treatment for 24 h at 37°C colonies turned to light yellow. Colonies in RHS group spent more time to cover the plate, and contained less aerial hyphae than those in Con group.

To find the response of cell structure to heat stress, mycelia were strained by CFSE and Congo red, and observed using CLSM (Figure 1B). Septa in mycelia from Con group were clear, while septa were not very clear after the mycelia were treated at 37°C for 8 h (HS_8_ group). After treated at 37°C for 24 h (HS group), septa could not be observed. The results indicated that septa were gradually degraded after heat treatment. The conformation of nucleus was changed with time after heat treatment. Intact big nucleus could be observed in mycelia from Con group, and the micronuclei appeared after treated for 8 h at 37°C (HS_8_ group). After treated at 37°C for 24 h, intact big nucleus disappeared, and the DNA was fragmented and distributed in the whole mycelia (Figure 1B).

Heat stress-induced oxidative damage could be measured by TBARS assay (Ghani et al., 2017). TBARS concentrations were 2.66 ± 0.28 nmol/g fresh weight (FW), 3.54 ± 0.27 nmol/g FW, and 3.04 ± 0.10 nmol/g FW in mycelia from Con group, HS group and RHS group, respectively (Figure 1C). TBARS concentration in HS group was significantly higher than that in Con group and RHS group. TBARS concentrations in RHS group was still significantly higher than that in Con group. The results indicated that heat treatment lead to heat stress-induced peroxidation and destruction in lipid membranes. When growth condition return to normal temperature, *H. marmoreus* mycelia partially repaired the oxidative damage in mycelia.

### Protein Identification and Quantification

**FIGURE 2:**
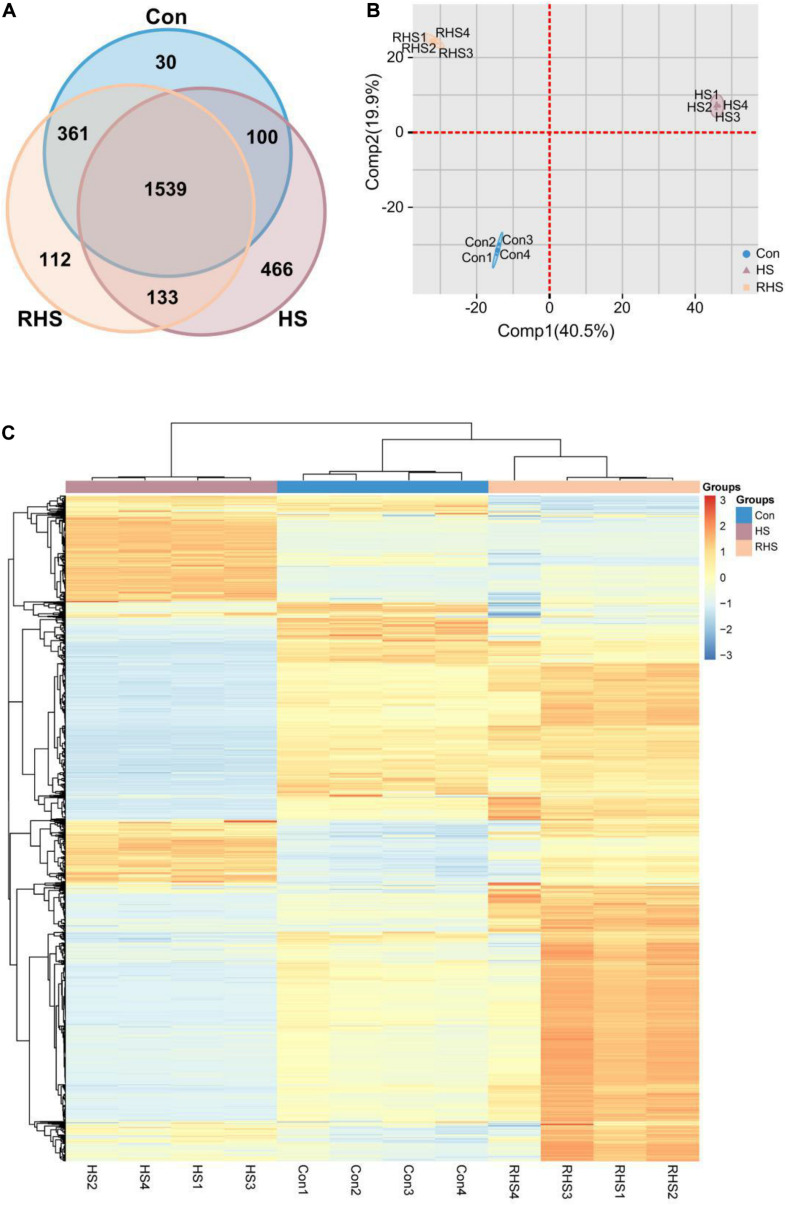
Venn diagram, PCA analysis and heat map analysis representation of the proteomic dataset of three groups. **(A)** Venn diagram of proteins identified in the proteomes of Con, HS and RHS groups. **(B)** PCA scores of the first two principal components based on individual proteomic data from Con, HS and RHS groups. **(C)** Heat map generated from 1539 proteins identified in all samples by hierarchical clustering using paired Euclidean distance.

Proteins were extracted from mycelia from Con group, HS group and RHS group, respectively, and subjected to label free quantitative proteomic analysis. A total of 2,741 proteins were identified in 12 runs of protein samples, and 2,030, 2,145, and 2,238 proteins were identified in Con group, HS group and RHS group, respectively (Figure 2A). Among the identified proteins, 30 proteins were unique to Con, 112 proteins were unique to RHS and 466 proteins were unique to HS (Figure 2A and Supplementary Table 1).

To investigate potential proteome differences between samples from different groups, principal component analysis (PCA) was performed. The first two principal components capture 60.4% of the variance in the dataset. Figure 2B shows the proportion of variance contributed by each sample to the first two components. The greatest component of variation in the data, Comp1, clearly differentiated HS group from other two groups, while both Con group and RHS group were located with negative Comp1 scores. A positive Comp2 score and negative Comp2 score discriminated Con group and RHS group. The order of samples was determined by heat map analysis. The result revealed that samples from the same group clustered in one cluster, respectively, and a division was observed between HS group and the other two groups (Figure 2C).

**FIGURE 3:**
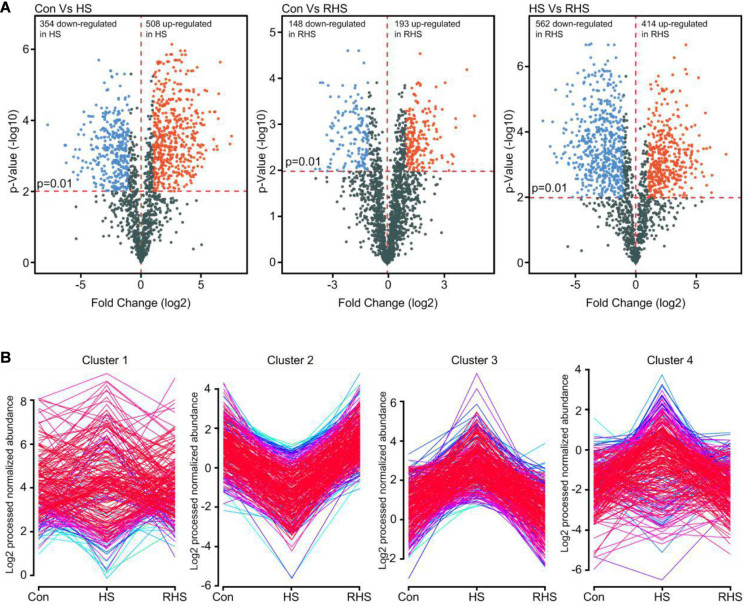
Analysis of DEPs of three proteome samples. **(A)** Volcano plot showing changes in protein expression from Con, HS and RHS groups. **(B)** Distribution profiles of DEPs divided in five major groups by Mfuzz clustering analysis.

A 2.0-fold change cut off and *p*-value BH < 0.01 were used to categorize differentially expressed proteins (DEPs). Compared with Con group, HS group was associated with 508 proteins that were upregulated and 354 proteins that were downregulated. Compared with HS group, 414 proteins were upregulated and 562 proteins were downregulated in RHS group. DEPs between Con group and RHS group were less than those between HS group and other groups. 193 proteins were upregulated in RHS group compared with Con group and 148 were downregulated (Figure 3A and Supplementary Tables 1, 2). Expression of 406 proteins were upregulated in HS group compared with both Con group and RHS group and 466 proteins were only identified in HS group. These 872 proteins were designated as HS-up DEPs. Expression of 313 proteins were downregulated in HS group compared with both Con and RHS group, and 361 proteins were not identified in HS but identified in both Con group and RHS group. These 674 proteins were designated as HS-down DEPs.

The DEPs expression profiles were conducted for Mfuzz clustering analysis to investigate the time-dependent protein expression pattern under heat stress (Kumar and Futschik, 2007). Two of the five clusters, cluster 2 and cluster 3, were easy to interpret. Cluster 2 contains proteins that were downregulated after heat treatment and increased to normal expression levels after recovery growth. In contrast, cluster 3 contains proteins that were upregulated after heat treatment and decreased to normal expression levels after recovery cultivation (Figure 3B). Proteins with continuously increasing or decreasing expression levels from Con group to HS group to RHS group were not detected with Mfuzz analysis.

### Functional Categorization Analysis

**FIGURE 4:**
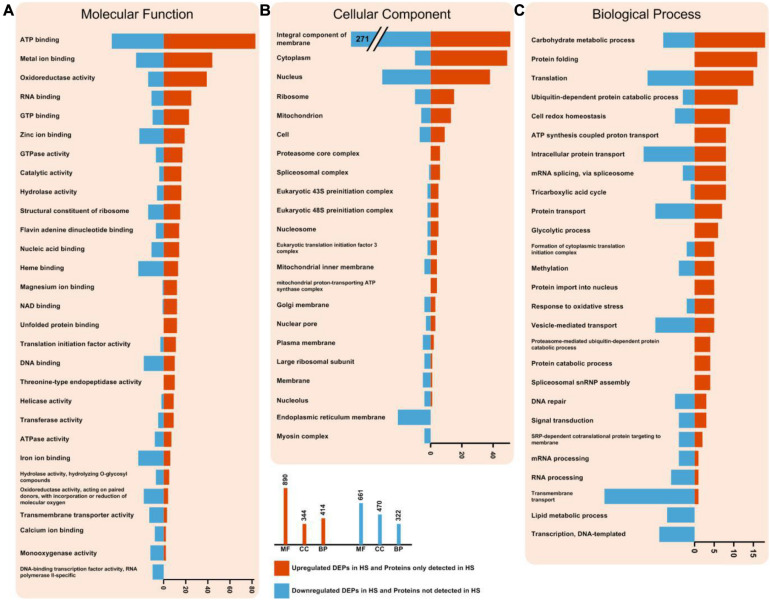
Enriched GO terms of HS-up DEPs and HS-down DEPs. **(A)** DEPs in molecular function (MF) term. **(B)** DEPs in cellular component (CC) term. **(C)** DEPs in biological process (BP) term. The statistics with more than 3 proteins at GO level 2 is shown in this figure. HS-up DEPs are shown in red, and HS-down DEPs are shown in blue.

To gain functional information about DEPs, GO annotations enrichment of HS-up DEPs and HS-down DEPs, which were classified into molecular function, cell components and biological process, were performed (Figure 4). The results of enrichment analysis showed that the distributions of proteins in HS-up DEPs and HS-down DEPs were different. Under the category of molecular function, HS-up DEPs were mainly found in the ATP binding, metal ion binding, oxidoreductase activity, RNA binding and GTP binding; while HS-down DEPs were mainly found in ATP binding, metal ion binding, heme binding, iron ion binding and zinc ion binding (Figure 4A). In the cellular component, HS-up DEPs were mainly enriched in integral component of membrane, cytoplasm, nucleus, ribosome and mitochondrion; while HS-down DEPs were mainly enriched in integral component of membrane, nucleus, endoplasmic reticulum membrane, ribosome and cytoplasm (Figure 4B). In biological process, HS-up DEPs were mostly related to carbohydrate metabolic process, protein folding, translation, ubiquitin-dependent protein catabolic process, and cell redox homeostasis; while HS-down DEPs were mostly related to transmembrane transport, intracellular protein transport, translation, vesicle-mediated transport and protein transport (Figure 4C).

**FIGURE 5:**
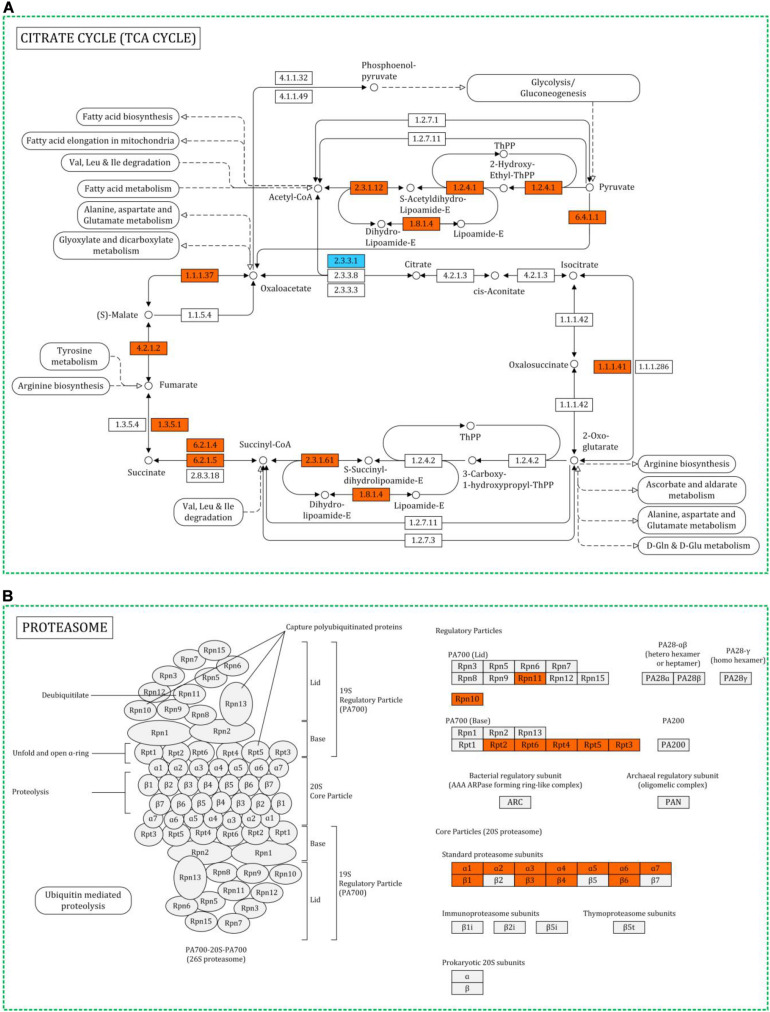
Representative metabolic pathway maps of differentially expressed proteins involved in TCA cycle **(A)** and proteasome **(B)** in KEGG. Red for upregulated proteins, blue for downregulated proteins.

The HS-up DEPs were associated with 100 specific KEGG pathways, which were mainly distributed in carbon metabolism (path: 01200), oxidative phosphorylation (path 00190), spliceosome (path: 03040), RNA transport (path: 03013), endocytosis (path: 04144), protein processing in endoplasmic reticulum (path: 04141), proteasome (path: 03050) (Figure 5A), glycolysis/gluconeogenesis (path: 00010), tricarboxylic acid cycle (TCA cycle) (path: 00020) (Figure 5A), respectively. The HS-down DEPs were associated with 92 specific KEGG pathways, which were mainly distributed in autophagy (path: 04138), protein processing in endoplasmic reticulum (path: 04141), protein export (path: 03060), glycerophospholipid metabolism (path: 00564), starch and sucrose metabolism (path: 00500), and N-glycan biosynthesis (path: 00510), respectively.

### Protein-Protein Interactions (PPI) for DEPs

**FIGURE 6:**
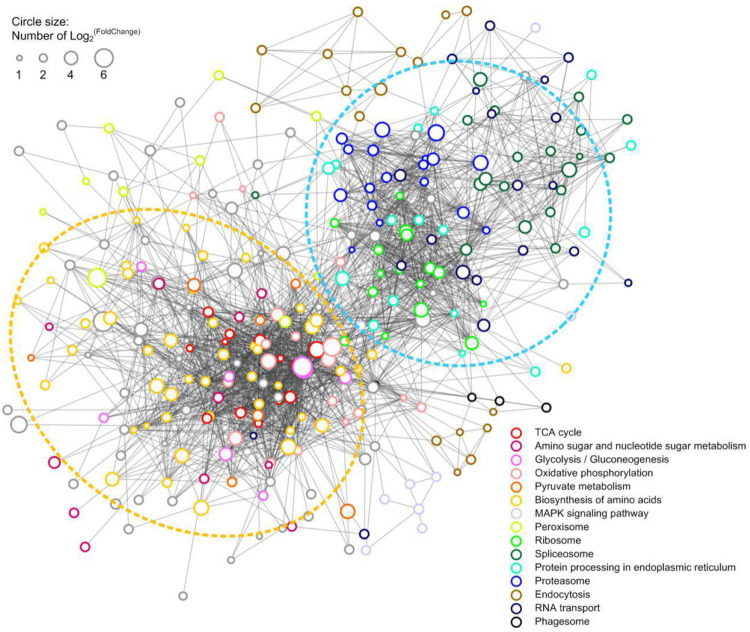
PPI network of the HS-up DEPs. 297 HS-up DEPs were searched for their PPI using the web resource STRING and characterized using Cytoscape. Lines represented interactions between proteins, and the size of circle denoted the fold change of the DEPs. The enriched KEGG functions of the DEPs were indicated with different color.

A PPI network of the upregulated DEPs in HS group was constructed to reveal how the HS-up DEPs are related to multiple interaction pathways. HS-up DEPs (311 proteins), with at least 10 DEPs in KEGG enrichment pathways, were subjected to PPI and network analysis using the web-based tool STRING. Among the 311 DEPs, 295 were included in the network and connected with each other. Based on the PPI analysis (Figure 6), one main interactive cluster was formed which contained two main sub-clusters. The sub-cluster in the yellow dashed circle was associated with TCA cycle, amino sugar and nucleotide sugar metabolism, glycolysis/gluconeogenesis, oxidative phosphorylation, pyruvate metabolism and biosynthesis of amino acids. The sub-cluster in the blue dashed circle was associated with ribosome, spliceosome, protein processing in endoplasmic reticulum, proteasome and RNA transport. The complicated PPI networks suggest that the physiological interaction of proteins in these processes may contribute to the global regulation of heat stress response in *H. marmoreus*.

### mRNA Expression of Selected Genes

**FIGURE 7:**
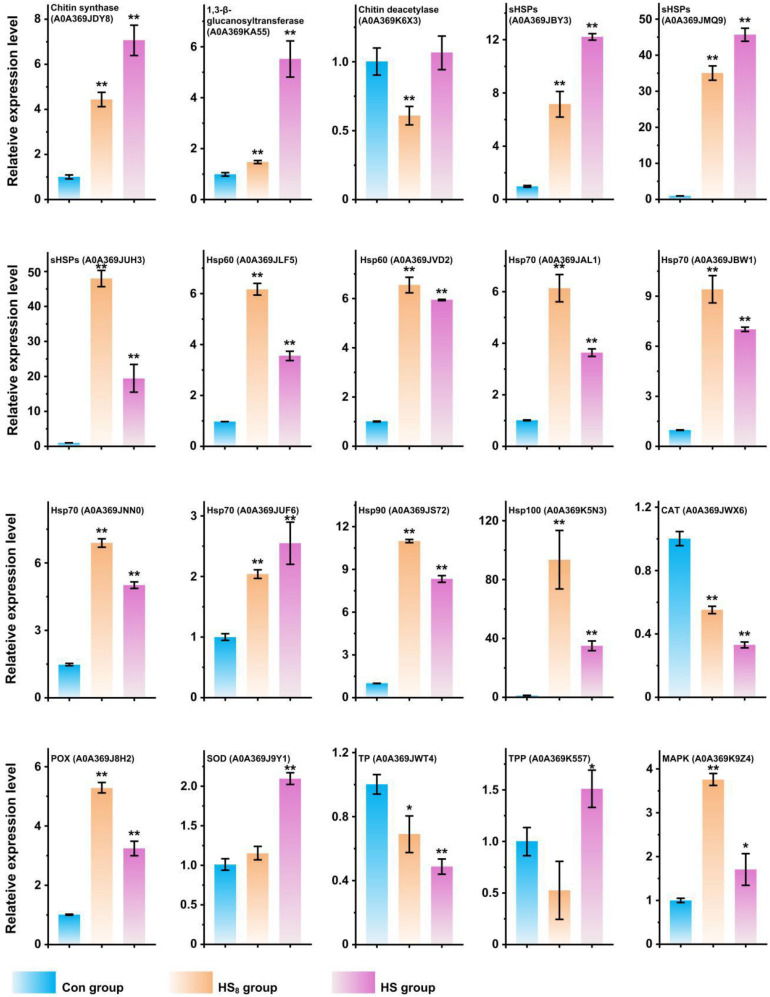
RT-qPCR analysis of transcriptional expression analysis of representative protein coding genes. The expression level of genes in Con group was set as 1. *t*-test was used to calculate statistical significance between HS group and Con group and between HS_8_ group and Con group. *0.01 ≦ *p* < 0.05; **p < 0.01.

To confirm the protein differences at the transcript level, the mRNA expressions of 20 differentially expressed key proteins were analyzed. The result showed that mRMA expression levels of 17 genes (85%) were upregulated (fold-change was more than 1.5-fold and *p*-value <0.05) in HS_8_ group or HS group compared with those in Con group, which exhibited the same trends at the mRNA and protein levels (Figure 7). Ten genes showed higher mRNA expression levels in HS_8_ than in HS group, indicating that mRNA expression level of these genes was upregulated in the early stage after heat treatment. One genes (Chitin deacetylase, A0A369K6X3) did not show significant difference in mRNA expression level after heat treatment. However, mRNA expression levels of two genes (10%), encoding one CAT (A0A369JWX6) and one TP (A0A369JWT4), significantly decreased after heat treatment. The expression of the two proteins may be regulated at the level of translation or post-translation modification.

## Discussion

Heat stress is an important adverse environmental stress that influences the growth and development of mushrooms. The vegetative growth period of *H. marmoreus* is about 80–90 days, which comprises about 80% of the whole cultivation period from seeding to harvest. This stage is vulnerable to heat stress, which can lead to substantial yield loss. Two typhoons slammed into Shandong province in 2018 and 2019, respectively. The corresponding damage of electric power facilities in mushroom producing areas in 2019 lead to temperature increase to ∼37°C about 24 h in *H. marmoreus* cultivation room. The cultivation factory suffered great loss. Therefore, understanding heat stress response mechanism and thereby producing transgenic strains with enhanced resistance to heat stress is necessary. Identification of genes/proteins responsive to heat stress is an essential first step toward understanding the molecular mechanisms. In the present study, we stimulated the heat stress condition (37°C for 24 h) and studied the heat stress response and recovery growth after heat stress by label-free proteomics analysis.

The volcano plot, PCA analysis and HCA analysis revealed that protein expression patterns were similar between Con group and RHS group. However, protein expression pattern of HS group was quite different from both Con group and RHS group. Mfuzz analysis further proved that the expression of most DEPs in HS group returned to the normal expression level in RHS group. Clearly, *H. marmoreus* mycelia induced differential protein expression pattern to respond heat stress, and returned to normal growth by eliminating this differential protein expression. Therefore, DEPs between Con group and HS group instead of DEPs between Con group and RHS group are essential for understanding of heat stress response mechanism in *H. marmoreus*.

### Autolysis and Apoptosis

CLMS images showed that, after treated at 37°C for 24 h, the septa of mycelia disappeared. This was caused by partial degradation of cell wall suggesting that mycelia underwent autolysis under heat stress. Heat induced autolysis was also observed in other fungi, the mycelia breakage and cytoplasmic inclusion leakage were observed in *L. edodes* and *Pleurotus tuber-regium* after treated at 40°C for 24 h or 12 h, respectively (Huang et al., 2011; Wang et al., 2018b). In this study, *H. marmoreus* mycelia were intact and cytoplasmic inclusion leakage was not observed indicating that 37°C is a mild heat stress temperature. Protein expression pattern was consistent with the experimental results. Polysaccharides such as chitin and β-(1,3) glucan are the core components of fungal cell wall (Gow et al., 2017). Chitin synthase is responsible for the synthesis of chitin, while β-(1,3) glucan is synthesized by glucan synthase complex using UDP-glucose as a substrate (Gow et al., 2017). Six chitin synthases were identified in this study. Three of them were not identified in HS group but identified in Con and RHS group, and two of them are downregulated under heat stress (Table 1). Two glucan synthase subunits were identified, and both of them were HS-down DEPs (Table 1). Chitin deacetylase and 1,3-beta-glucanosyltransferase play a major role in cell wall remodeling and degradation (Mouyna et al., 2013; Gow et al., 2017). Two chitin deacetylases and one 1,3-beta-glucanosyltransferase were identified, and all of them were HS-up DEPs (Table 1). Clearly, protein expression pattern revealed that the cell wall softening and degradation processes were upregulated, while cell wall synthesize processes were inhibited under heat stress. Degradation of septa might be good for material transportation between hypha cells, and cell wall degradation could also provide carbon sources for other cellular processes.

**TABLE 1:** Expression of some differential expression proteins. ∞ indicates that the denominator in the fraction is zero.

UniProt ID	Fold change			Description
	HS/Con	HS/RHS	RHS/Con	
**Chitin synthase**				
A0A369IZU7	0.871	0.360	2.416	Chitin synthase
A0A369JDX0	0.448	0.077	5.823	Chitin synthase 8
A0A369JZJ9	0	0	1.527	Chitin synthase 1
A0A369JJ99	0.133	0.111	1.200	Chitin synthase export chaperone
A0A369JK46	0	0	2.776	Chitin synthase
A0A369JDY8	0	0	2.045	Chitin synthase 8
**Alpha-1,3-glucan synthase**				
A0A369JFX8	0	0	1.938	alpha-1,3-glucan synthase mok13
A0A369K433	0.122	0.075	1.637	1,3-beta-glucan synthase component FKS1
**Chitin deacetylase**				
A0A369K6X3	∞	∞	NA	Chitin deacetylase
A0A369JT12	0.801	5.009	0.160	Chitin deacetylase
**1,3-Beta-glucanosyltransferase**				
A0A369KA55	18.214	7.002	2.601	1,3-Beta-glucanosyltransferase
**Caspase**				
A0A369K220	∞	3.383	∞	Metacaspase-1B
**sHSPs**				
A0A369JGZ2	∞	∞	NA	Small heat shock protein
A0A369JBY3	29.723	17.794	1.670	Heat shock protein, mitochondrial
A0A369KA26	∞	21.925	∞	Heat shock protein 16
A0A369JFM9	185.153	∞	0	Heat shock protein 16
A0A369JMQ9	∞	203.070	∞	Heat shock protein 16
A0A369IY45	∞	∞	NA	Small heat shock protein C4
A0A369JUH3	19.897	19.639	1.013	DnaJ subfamily B member 4
**Hsp60**				
A0A369JLF5	14.750	53.708	0.275	Heat shock protein sti1
A0A369JVD2	30.839	20.842	1.480	Heat shock protein 60, mitochondrial
**Hsp70**				
A0A369JAL1	20.737	19.498	1.064	Heat shock protein Hsp88
A0A369JBW1	6.497	18.975	0.342	Glucose-regulated
A0A369JNN0	7.259	5.785	1.255	Heat shock protein HSS1
A0A369JUF6	18.307	25.628	0.714	Heat shock protein sks2
A0A369K2K3	9.805	13.124	0.747	Heat shock protein (HmHsp70)
A0A369KH52	0.611	0.325	1.883	Heat shock protein 70
A0A369K2Q0	∞	∞	NA	Heat shock protein 78, mitochondrial
**Hsp90**				
A0A369JXH4	1.922	1.088	1.766	Heat shock protein 90
A0A369JS72	2.794	3.070	0.910	Heat shock protein 90
**Hsp100**				
A0A369JUV8	0.024	0.109	0.218	Heat shock protein 12A
A0A369K5N3	39.700	42.046	0.944	Uncharacterized protein
**CAT**				
A0A369JWX6	2.733	5.666	0.482	Catalase
**POX**				
A0A369JL11	2.051	3.953	0.519	Peroxidase 2
A0A369J8H2	2.705	8.860	0.305	Peroxidase
A0A369K511	0.817	1.100	0.743	Glutathione peroxidase
**SOD**				
A0A369K670	0.525	0.379	1.387	Superoxide dismutase
A0A369J9Y1	9.188	18.260	0.503	Superoxide dismutase
A0A369JBU6	∞	20.806	∞	Superoxide dismutase [Cu-Zn]
**TP**				
A0A369JWT4	2.113	4.396	0.481	Trehalose phosphorylase
**TPS**				
A0A369JTP6	0.624	0.972	0.642	Trehalose-phosphate synthase
**TPP**				
A0A369JFC7	1.009	0.880	1.146	Trehalose-6-phosphate phosphatase
A0A369K557	1.832	3.273	0.560	Trehalose-6-phosphate phosphatase
**MAPK**				
A0A369K4T8	1.140	1.091	1.045	Mitogen-activated protein kinase
A0A369K9Z4	3.744	2.591	1.446	Mitogen-activated protein kinase
A0A369K7W7	0	0	0.211	Mitogen-activated protein kinase
A0A369JVM9	∞	∞	NA	Mitogen-activated protein kinase
**MAPKK**				
A0A369J2M8	∞	∞	NA	MAP kinase kinase
A0A369JR52	∞	∞	NA	MAP kinase kinase
**MAPKKK**				
A0A369K859	1.131	2.739	0.413	MAP kinase kinase kinase

Apoptosis was one of the typical stress response in fungi, which could triggered by different stimuli (Sharon et al., 2009). Song et al. reported that heat stress induced the apoptosis cell death in two *Pleurotus* species, nuclear condensation and DNA fragmentation in hypha cells were observed (Song et al., 2014). Nucleus fragmentation was also observed in this study, indicating that apoptosis was induced by heat stress in *H. marmoreus* cells (Phillips et al., 2003; Sharon et al., 2009). Besides, one metacaspase (A0A369K220) was identified in HS group but not identified in Con group, and the expression of this metacaspase in HS group was 2.383-fold higher than that in RHS group. TBARS analysis indicated the ROS accumulation in *H. marmoreus* mycelia. ROS was reported as apoptosis trigger molecules while caspases are located at the end of the apoptotic signal transduction chain. Therefore, apoptosis in *H. marmoreus* might be mediated by ROS and caspases activation.

### Proteins Related to Protein Folding and Degradation

HSPs are representative molecular chaperons which play critical roles in regulating organism growth and development as well as prevent the formation of misfolded protein structures, rescue the previously aggregated or denatured proteins when cells are exposed to stress, such as high temperature (Richter et al., 2010). Induction of a series of highly conserved HSPs is one of the most well characterized heat stress response. Proteomic or transcriptome studies have revealed that HSPs are upregulated in mushroom mycelia under heat stress, showing that HSPs play important role in the resistance of heat stress in mushrooms (Chen et al., 2017; Wang et al., 2018b; Zou et al., 2018). HSPs are classified as small HSPs, Hsp60, Hsp70, Hsp90 and Hsp100 based on molecular weight (Richter et al., 2010). All kinds of HSPs have been reported playing important roles in heat stress response. In this study, the expression of seven small HSPs (sHSPs), two Hsp60s, six Hsp70s, one Hsp90 and one Hsp100 were upregulated, while only one Hsp100 protein expression was downregulated after heat treatment (Table 1).

sHSPs comprise the most widespread family of molecular chaperones, which protect proteins from irreversible aggregation by reversible interactions with a chaperone (Richter et al., 2010). The sHSP gene, *LeDnaJ*, silence mutants of *L. edodes* have defects in mycelial growth under heat stress (Wang et al., 2018a). Here, a sHSP (A0A369JUH3) shows highest sequence identity (65.2%) with LeDnaJ in *H. marmoreus* and the expression of A0A369JUH3 also upregulated (19.897-fold) under heat stress. Besides, the expression of two sHSPs increased by 28.723-fold and 184.153-fold under heat stress, respectively, and 4 sHSPs were only identified in HS group but not identified in Con group. sHSPs in *H. marmoreus* might have variety functions in alleviating heat stress.

In this study, two Hsp60s (A0A369JLF5 and A0A369JVD2) were upregulated under heat stress and then returned to normal levels after recovery cultivation. Similar result was observed in *P. ostreatus* (Zou et al., 2018). Based on the previously studies (Martin et al., 1992), chaperons of Hsp60 family might stabilize preexisting proteins under stress conditions in *H. marmoreus*.

Hsp70 family represents the most highly conserved HSPs, prevents the aggregation of unfolding proteins, and can even refold aggregated proteins under stress (Kiang and Tsokos, 1998). In our previous report, one Hsp70 (A0A369K2K3) gene, *hmHsp70*, was cloned from *H. marmoreus* and cloned into tobacco. The transgenic tobacco showed enhanced resistance to lethal temperature (Xu et al., 2020). Similar result was observed in this study, the expression of HmHsp70 increased by 8.805 fold and then decreased to 0.747 fold after recovery cultivation. The expressions of five other Hsp70s were also upregulated under heat stress. The expression of Hsp70s might confer thermotolerance to *H. marmoreus*, which makes them promising target proteins in thermotolerance study.

Hsp90 is an ATP dependent molecular champerone which distributed in all organisms. Hsp90 can regulate intracellular signal transduction through folding and activation of proteins that are prone to misfolding. Kimura et al. reported that Hsp90 mutant of *Saccharomyces cerevisiae* was sensitive to high temperature (Kimura et al., 1994). A0A369JS72 in *H. marmoreus* shows the highest sequence identity with the Hsp90 in *S. cerevisiae* and also upregulated under heat stress. The results confirmed that Hsp90 is involved in heat stress response in *H. marmoreus*, which might involved in apoptosis signal transduction.

It has been reported that Hsp100 play crucial thermotolerance roles in fungi (Richter et al., 2010). In *S. cerevisiae*, Hsp104 could reactivate denatured proteins and therefore enhance cell survival after exposure to extreme heat (Glover and Lindquist, 1998). The thermotolerance defect in *S. cerevisiae hsp104* mutant could be complemented by overexpression of an Hsp100 gene, *PsHsp100*, from *Pleurotus sajor-caju* (Lee et al., 2006). In *H. marmoreus*, A0A369K5N3 showed the highest sequence identity (76.6%) with PsHsp100. The expression of A0A369K5N3 increased 38.7-fold under heat stress and decreased to 0.9-fold after recovery cultivation. A0A369K5N3 in *H. marmoreus* may cooperate with other chaperones to alleviate the protein denaturation caused by heat stress. In summary, *H. marmoreus* induced a series chaperons to protect preexisting proteins, help the synthesized proteins folding and rescued the denatured proteins under heat stress.

Heat stress can lead to protein misfolding and aggregation (Richter et al., 2010). Although, HSPs could prevent this precess, there were still some denatured proteins which could not be rescued. These proteins need to be cleared and reused. Proteasome plays a catalytic part in the ubiquitin-proteasome pathway, which could degraded the denatured proteins. Huang et al. reported the upregulation of proteasome in *P. tuber-regium* under heat stress (Huang et al., 2011). Similar results were observed in this study, several subunit of proteasome were upregulated under heat stress in *H. marmoreus* (Figure 5B). The proteasome could prevent the accumulation of non-functional and potentially toxic proteins and provide amino acids for proteins systhesis.

### Protein Related to ROS Scavenging

ROS are typical signal molecules in response to heat stress in all organisms. The accumulation of ROS was also reported in several mushroom mycelia under heat stress (Liu et al., 2018c; Zou et al., 2018; Lei et al., 2019). High concentration of ROS can lead to the disruption of redox homeostasis and induce oxidative stress damaging such as protein degradation, DNA damage, membrane peroxidation, and apoptosis (Zuo et al., 2015). Membranes are rich in unsaturated fatty acid which are potentially susceptible to ROS attack, therefore, membranes are particularly susceptible to oxidative damage by ROS. Lipid oxidation of membrances produce many products with aldehydes (such as malondialdehyde) prominent among them. TBA can reacts with aldehydes to produce a pink-colored dimeric compound, therefore TBARS analysis was the most common used method to measure lipid oxidation (Slimen et al., 2014; Ghani et al., 2017). The TBARS concentration of HS group was significantly higher than that in Con or RHS group, indicating that the ROS was accumulated and lipids were damaged by ROS under heat stress in *H. marmoreus* mycelia.

In order to prevent the damage caused by ROS and maintain the steady-state concentration of ROS, cells induce antioxidant system allowing the scavenging and neutralization of ROS generated. The main antioxidant defense system is composed by antioxidant enzymes including catalase (CAT), superoxide dismutase (SOD) and peroxidase (POX). SOD could transform superoxide anions, which is the precursor of most ROS, to hydrogen peroxide (H_2_O_2_). H_2_O_2_ is then detoxified by CAT and POX to form water (Slimen et al., 2014). In this study, one CAT, three POXs and three SODs were identified, five of them are upregulated or only expressed under heat stress (Table 1). No protein was downregulated under heat stress. Similar results were reported in other mushrooms. The expressions of two CAT genes, *Po-cat1* and *Po-cat2*, were upregulated under heat stress in *P. ostreatus* (Wang et al., 2017). The enzyme activity of CAT, POX and SOD increased in *Ganoderma lucidum* mycelia after heat treatment (Liu et al., 2018c). The results indicated that upregulated ROS scavengers played important roles in response to heat stress in *H. marmoreus.* Previous studies proved that ROS scavenging is one of the effective way to increase thermotolerance to mushroom mycelia. Adding *N*-acetyl cysteine and vitamin C could reduced the malondialdehyde level and recovered mycelial growth under heat stress in *P. ostreatus* (Lei et al., 2019). Therefore, upregulated ROS scavenging proteins identified in this study can be used as candidate target to improve thermotolerance of *H. marmoreus* strain.

### Proteins Related to Carbon and Energy Metabolism

Heat stress responses such as protein biosynthesis, folding, activation and degradation and ROS scavenging require a great mount of energy. Therefore, ATP is essential for mycelia to respond heat stress. Here, nine ATP synthase subunits were identified. Two of them were only identified in HS group but not identified in Con group, and the other seven proteins were all highly upregulated under heat stress (Table 1). This is consistent with the energy requirement under heat stress. For cells, the main way to generate ATP is based on TCA cycle. Figure 5A showed that thirteen proteins involved in TCA cycle were upregulated, while only one was downregulated. The results suggested that the mycelia attempted to generate more energy to cope with heat stress by inducing TCA cycle and ATP synthesis processes. Similar results were reported in cucumber leaves, proteins involved in ATP synthesis and TCA cycle were upregulated under heat stress (Xu et al., 2018). However, TCA cycle was inhibited in *P. ostreatus* after 48 h of heat stress treatment at 40°C (Zou et al., 2018). The temperature and treated time for *P. ostreatus* were higher than those in this study. It might due to that the energy synthesis system was seriously damaged in that conditions.

### Protein Related to Trehalose Metabolism

Increased level of trehalose is a response to stress conditions, such as high temperature, dehydration, oxidative stress and glucose starvation, for fungi (Kong et al., 2012b; Liu et al., 2016, 2019; Zhao et al., 2018; Lei et al., 2019). Lei et al. reported that the accumulated trehalose in *P. ostreatus* could promote the recovery growth after heat stress, and trehalose production was partly induced by ROS accumulation (Lei et al., 2019). The induced trehalose was also observed in other mushrooms such as *F. velutipes*, *P. eryngii* and *Pleurotus pulmonarius* (Kong et al., 2012b; Liu et al., 2016, 2019). Two main trehalose synthesis pathways have been studied in mushrooms. The most common pathway involved two enzymes: trehalose-6-phosphate synthase (TPS) catalyzes the synthesis of trehalose-6-phosphate from glucose-6-phosphate and UDP-glucose, and trehalose-6-phosphate phosphatase (TPP) transform trehalose-6-phosphate to trehalose (Eleutherio et al., 2015; Zhao et al., 2018). One TPS and two TPPs were identified in this study (Table 1); while none of the three enzymes were upregulated or downregulated under heat stress. The other pathway was catalyzed by trehalose phosphorylase (TP), which could synthesize/degrade glucose-1-phosphate and glucose to/from trehalose (Han et al., 2003; Eleutherio et al., 2015). Han et al. firstly cloned a TP gene, *PsTP*, from *P. sajor-caju*, and proved its function in yeast (Han et al., 2003). A0A369JWT4 shows the highest sequence identity with PsTP, and the expression of A0A369JWT4 increased by 1.113-fold under heat stress compared with Con group and decreased to 0.481-fold in RHS group. The results confirmed that trehalose accumulation might took part in heat response in *H. marmoreus*. Although trehalose could be synthesized though TPS and TPP pathway under normal or heat stress conditions, TP might be crucial for trehalose synthesis/degradation regulation under heat stress in *H. marmoreus*.

### Protein Related to MAKP Signal Pathway

MAPK signaling pathway, firstly elucidated in *S. cerevisiae*, plays important roles in response to a broad variety of biotic and abiotic stresses, including heat stress. MAPK cascade, including MAPKs, MAPK kinases (MAPKKs) and MAPKK kinases (MAPKKKs), propagates stress signals, modulate cellular processes, and is controlled by regulatory factors (Smith et al., 2010; Lee et al., 2019). In *P. ostreatus*, a MAPK was increased by 2.0-fold under heat stress (Zou et al., 2018). Similar results were also reported in *L. edodes* (Wang et al., 2018b). Here, four MAPKs, two MAPKKs and one MAPKKK were identified. Two MAPKs and two MAPKKs are upregulated or only expressed in HS group, and then the expression level returned to normal level after recovery growth. Only one MAPK was downregulated (Table 1). The results indicated that the MAPK cascade played important role in transmitting heat stress signal in *H. marmoreus*.

## Conclusion

In this work, we performed label-free proteomics analysis to investigate the mechanism of heat stress response in mycelia of *H. marmoreus.* Experiments and data analysis of mycelia with different treatments confirm enhanced expression levels of proteins with signal transduction, energy production, cell wall degradation, protein folding and degradation, trehalose synthesis and ROS scavenging. The results indicated that proteotoxicity and ROS are the main harmful factors to *H. marmoreus* mycelia under heat stress, and *H. marmoreus* induced a series of cellular processes to protect cells against these harmful factors together with energy production to fulfill the energy demands. Information provided by this study can help us understand the heat stress response mechanism of *H. marmoreus.* Nevertheless, the signal transduction processes and the regulation mechanism of HSPs in *H. marmoreus* under heat stress remain unclear. The key proteins identified by proteomics analysis have been successfully used to rebuild and enhance the thermotolerance of mushrooms (Wang et al., 2018b; Xu et al., 2020). Therefore, this study may be helpful for the breeding of heat-resistance *H. marmoreus* strains.

## Data Availability Statement

The data presented in the study are deposited in the ProteomeXchange repository, accession number PXD017654.

## Author Contributions

HY and LX conceived and designed the project, wrote the manuscript. HY, LG, and LX performed the experiments, contributed reagents and materials, and analyzed data. All of the authors have read and approved the manuscript.

## Conflict of Interest

The authors declare that the research was conducted in the absence of any commercial or financial relationships that could be construed as a potential conflict of interest.

## Footnotes

1 http://geneontology.org/

## Abbreviations

ROS: reactive oxygen species
HSPs: heat shock proteins
LC-MS: liquid chromatography-tandem mass spectrometry
CFSE: carboxyfluorescein succinimidyl ester
CLSM: confocal laser scanning microscope
TBARS: thiobarbituric acid reactive substances
FW: fresh weight
PCA: principal component analysis
DEPs: differentially expressed proteins
Con: control
HS: heat stress
HS_8_: heat stress at 37 for 8 hours
RHS: recovery after heat shock
TCA cycle: tricarboxylic acid cycle
PPI: protein-protein interactions
sHSP: small HSP
CAT: catalase
SOD: superoxide dismutase
POX: peroxidase
H_2_O_2_: hydrogen peroxide
TPS: trehalose-6-phosphate synthase
TPP: trehalose-6-phosphate phosphatase
TP: trehalose phosphorylase
MAPK: mitogen-activated protein kinase
MAPKK: MAPK kinase
MAPKKK: MAPKK kinase
PDA: potato dextrose agar
BH: Benjamini-Hochberg
qRT-PCR: quantitative real-time PCR
GO: gene ontology
KEGG: Kyoto Encyclopedia of Genes and Genomes
STRING: Serach Tool for the Retrieval of Interacting Genes/Proteins
HS-up DEPs: DEPs that upregulated in HS group compared with both Con and RHS group and DEPs that only identified in HS group
HS-down DEPs: DEPs that downregulated in HS group compared with both Con and RHS group and DEPs that identified in both Con and RHS group but not identified in HS group.
